# Honorary Degrees

**Published:** 1868-09-15

**Authors:** 


### *Honorary Degrees.*

When the Western and Nashville Journals get through their *unwilling* noddles the idea that HONORARY DEGREES have, from time immemorial, been conferred upon those who have especially distinguished themselves in medicine, as writers, discoverers or practitioners, it will be time enough to reply to their discreditable slurs upon certain gentlemen who have recently been selected as recipients of this honor by "a certain medical college." The fact of previous graduation or non-graduation, of course, has nothing to do with it.

*Our Amiable Contemporary* out West will receive suitable attention soon.

"*What's in a Name?*" An article under this heading is on file for insertion soon.

